# The Sequelæ of Suppurative Otitis Media

**Published:** 1890-06

**Authors:** F. H. Edgeworth

**Affiliations:** Physician to the Bristol Hospital for Sick Children and Women; Scholar of Caius Coll., Cambridge; late Medical Tutor, Bristol Medical School


### THE SEQUELÆ OF SUPPURATIVE OTITIS MEDIA.

BY

F. H. EDGEWORTH, B.A., M.B. (Cantab), B.Sc. (Lond.),

*Physician to the Bristol Hospital for Sick Children and Women; Scholar of Caius Coll., Cambridge; Late Medical Tutor, Bristol Medical School.*

(*Continued from page* 28.)

8 and 9. *Brain abscess.*—The importance of middle-ear disease as a cause of brain abscess is very great; for it is estimated that from 40% to 50% (Gowers, 42%; Barker, 50%) of all brain abscesses which occur without external wound are due to suppurative otitis media. As to the position which an abscess in the brain from ear disease may occupy, statistics have been compiled by Barr from seventy-six cases, showing that—

73% occur in the cerebrum.
19% ,, ,, cerebellum.
4% ,, in both cerebrum and cerebellum.
3% ,, in the pons.
1% ,, ,, crus cerebelli.

Again, in 87% of brain abscesses from ear disease, the abscess is single. Hence, practically, we have to deal with either a cerebral or a cerebellar abscess. Such an abscess is always on the same side of the brain as the ear disease; and a cerebral abscess is almost invariably in the temporo-sphenoidal lobe, whilst a cerebellar abscess is in the lateral lobe of the cerebellum.

The prior ear disease is generally chronic, has often lasted several years, and the determining factor is often deficient drainage. Abscess, then, is usually preceded by, although not dependent on, caries and necrosis of the temporal bone. In rare cases, however, brain abscess occurs in acute suppurative otitis media; and then the abscess is very often acute and very difficult to diagnose. Barker is of opinion that the acute non-fœtid brain abscess is due mostly to pathogenic cocci, which form the characteristic virus of acute suppurative otitis media; whilst in the chronic fœtid abscess, as in the preceding chronic otitis, the virus is almost solely of saprophytic bacilli.

*Temporo-sphenoidal abscess.*—A temporo-sphenoidal abscess is usually subcortical, arising within the lobe in the white substance, the cortex being little if at all changed. The exact path by which inflammatory changes pass into the lobe is not definitely known. There is a direct means of communication—veins pass from the white substance of the temporo-sphenoidal lobe to the superior petrosal sinus, to the dura-mater over the tympanic roof, and to the neighbourhood of the squamopetrous suture, whilst veins pass to these places from the middle ear. Hence it is supposed that inflammation spreads inwards by means of a retrograde phlebitis; and in some cases of temporo-sphenoidal abscess there have been found old clots in the superior petrosal sinus. But, as this is rare, another and perhaps more probable suggestion is, that the inflammation spreads inwards along the perivascular lymphatics of these veins. A third path has been suggested, by the perivascular lymphatics of the internal carotid artery; for this sends twigs to the middle ear. This view is probably not true of the great majority of cases; for a cerebral abscess from ear disease is almost invariably in the temporo-sphenoidal lobe, and not in any other portion of the cerebral area supplied by the internal carotid. Whatever the exact path, there arises a local septic inflammation in the lobe; leucocytes are poured out; nerve-elements undergo disintegration; red softening passes into an abscess. Such an abscess may be of any size, from that of a pea to one filling up the whole of the temporo-sphenoidal lobe. Usually, however, its size varies from that of a walnut to a hen's egg.

A second method of formation of a temporo-sphenoidal abscess is by a localised suppurative meningitis—such may be called subdural, in contradistinction to the subcortical form. Inflammation spreads through the tympanic roof; the dura becomes adherent; inflammation progresses through this to the pia-arachnoid. If such an inflammation take place slowly, adhesions may be formed round, so that the meningitis does not become general. The resulting abscess will have one wall formed by the dura mater; the other by the more or less necrosed brain-tissue of the temporo-sphenoidal lobe. Such an abscess, although different in its method of formation, will produce the same results as does that which begins within the cortex. On operation, the distinction between the subdural and subcortical forms can generally be made, but not always; for an abscess which begins subcortically may, in its enlargement, destroy the superjacent cortex.

The abscess which begins as a circumscribed leptomeningitis will, by reason of its method of formation, be more likely to be associated with general meningitis or sinus-thrombosis than will an abscess which begins subcortically.

The eventual fate of a temporo-sphenoidal abscess is to cause death, either by extensive softening taking place around it, or, more generally, by its enlarging and bursting into the meninges or lateral ventricle, and so setting up a rapidly fatal meningitis. Fagge estimates that five-sevenths of all temporo-sphenoidal abscesses end by rupture into the lateral ventricle.

The symptoms produced by temporo-sphenoidal abscess depend, in great measure, on its rate of enlargement. For descriptive purposes we may distinguish the following varieties: latent, slowly enlarging, rapidly enlarging, ruptured, abscess.

Whether or not an abscess may always remain latent is doubtful. Cases, in which well-marked symptoms have subsequently arisen, are sometimes preceded by a stage, often prolonged, in which there have been mental hebetude, slight headache, malaise, and occasional vomiting.

By far the most important stage, from a clinical point of view, is that in which a temporo-sphenoidal abscess is slowly enlarging and causing increased intracranial pressure. The results of increased intracranial pressure are extremely characteristic.

The *intelligence* is generally blunted. In several cases there has been noted "slow but perfect cerebration;" *i.e.*, mental processes are performed correctly, but very slowly. As the pressure increases, torpor and drowsiness come on —this passes in the terminal stages into coma and death. *Vomiting* is extremely characteristic—it has all the signs of being "cerebral;" *i.e.*, is without obvious cause, occurs independently of meals, and irregularly, often in paroxysms, and is generally without nausea. It is often associated with exacerbations of the headache. *Slow pulse* is a most significant symptom, more so perhaps than any other. *Headache* is always present; but its position is by no means always characteristic. In temporo-sphenoidal abscess it may be occipital or frontal, or general, or may spread from the region of the ear over the parietal bone. *Tenderness* on percussion of the skull, over a suspected abscess, is of much more value, especially when pain is not spontaneously complained of in that position.

These symptoms—blunted intelligence, torpor or coma, vomiting, slow pulse—are sufficient to enable one to diagnose a brain abscess in a case of otorrhœa: and if there are no signs characteristic of either temporo-sphenoidal or cerebellar abscess, the former lobe should be first explored; and if no pus be found there, then the latter. The temporo-sphenoidal lobe should be first explored; for brain abscess from ear disease is, as shown above, four times as common there as in the cerebellum.

There are, further, often present certain characteristic symptoms, the cause of which is uncertain or disputed, but the importance of which, when occurring, is great.

*Optic neuritis* may, as stated above, be present in cases of ear disease, with a total absence of all other cerebral symptoms. It may be present in cases of meningitis, and in cases of sinus-thrombosis. Hence its presence is not pathognomonic of abscess. It, however, most usually, although not always, occurs, and in some cases has been most marked in the opposite eye. The *temperature* in cases of slowly-enlarging abscess is normal or subnormal. This most important symptom is generally thought to be due to increased intracranial pressure. By Barker it is held to be caused by the products of the saprophytic bacilli so characteristic of these slowly-enlarging fœtid brain abscesses.

Barker has also pointed out the importance of two other symptoms—*rapid wasting* and *constipation*. The importance of the former is, that it occurs without any pyrexia to account for it; it is often associated with a muddy colour of the skin. Constipation, often marked, is most characteristic of uncomplicated abscess or meningitis. If diarrhœa exists, some septic infection has taken place; *i.e.*, complications of the ear disease, other than meningitis and brain abscess, have occurred.

In many cases the temporo-sphenoidal abscess will, as it slowly enlarges, exert pressure on neighbouring motor and sensory convolutions: the effects, more particularly the motor, though they be little marked, are extremely characteristic. Now, the temporo-sphenoidal lobe is only separated by the posterior limb of the fissure of Sylvius from the posterior end of the 3rd frontal gyrus and from the lower ends of the pre- and post-central gyri. Hence, as a temporo-sphenoidal abscess enlarges, it may press on those gyri, on the posterior end of the 3rd frontal, causing, when on the left side, motor aphasia; on the basal ends of the pre- and post-frontal gyri, causing paresis or paralysis of the opposite half of the tongue. If the abscess still further enlarge, it may exert pressure on the posterior end of the 2nd frontal gyrus, causing paresis of the opposite half of the tongue, and on the middle third of the pre-central gyrus, causing paresis of the opposite fingers, hand, wrist, forearm, arm, and shoulder muscles. There is no instance recorded of a temporo-sphenoidal abscess having affected the leg-centres.

*Sensory defects*, from pressure on neighbouring sensory convolutions, are much rarer. This is probably due to the abscess tending to enlarge forwards rather than upwards and backwards. Pressure on the posterior end of the superior temporo-sphenoidal gyrus will cause transitory deafness of the opposite ear; and if on the left side, *verbal deafness*, with resulting *amnesic aphasia* (of this only two instances are recorded). Pressure on the occipital lobe will produce *homonomous hemianopsia*, affecting the lateral halves of the retinæ on the same side as the lesion (of this only one instance is recorded).

Affections of the oculo-motor nerves sometimes occur, due to the pressure of the temporo-sphenoidal abscess on them as they lie round the cavernous sinus on the inner side of the anterior extremity of the lobe. Mydriasis, ptosis, paresis of the internal, superior, and inferior recti, and of the inferior oblique, result from pressure on the 3rd nerve; paresis of the superior oblique, from pressure on the 4th nerve. The 6th nerve, from its intracranial course, is less liable than the 3rd or 4th to be pressed upon; but this sometimes happens, paresis of the external rectus resulting.

As to the significance of these symptoms of local pressure, it may be said that their absence, in the presence of the above-described symptoms of general increased intracranial pressure, is of no negative value, but that their presence, even though slightly marked (the paralyses are seldom absolute), together with general symptoms, is absolutely characteristic of a temporo-sphenoidal abscess pressing on these convolutions or nerves.

*Acute temporo-sphenoidal abscess* will give rise, not only to symptoms of increased general and local intracranial pressure, but also to those due to inflammation of the brain-tissue round the abscess, and these may obscure the former. In addition to the paresis of the opposite half of the body, there will be convulsions of the same parts. And, together with these signs of local irritation, there will be those due to the inflammation: the temperature is high, with delirium and possibly general convulsions. Such a condition is with difficulty, if at all, diagnosed from general meningitis. The points which may, in some cases, determine a diagnosis of abscess are: the absence of any sign of basal meningitis, but the presence of signs of irritation of, and pressure on, the motor convolutions of one side of the brain only, and possibly the presence of a slow pulse; for this may be present, from increased intracranial pressure, even though the temperature is high. Such points may determine a diagnosis in favour of abscess, and so lead to operation which would probably save life in a case of abscess, whilst it could do no harm in a case of general septic meningitis, which is inevitably fatal.

Meningitis, however may co-exist, with abscess; if it is independent of it from the first, diagnosis is impossible. Meningitis resulting from rupture of an abscess could only be diagnosed if there were a history pointing to the previous presence of a slowly-enlarging abscess.

A bare outline need only be given of the operation for temporo-sphenoidal abscess. In a few cases there has been disease of the overlying bone, with a sinus leading down to the abscess; and Barker thinks these have been for the most part cases of extradural abscess. Almost invariably there is no such external sign of its presence.

The temporo-sphenoidal lobe is readily mapped out on the skull by Reid's lines. Authorities are not exactly agreed as to the best position within this area for trephining. Thus, Macewen trephines vertically above the meatus, whilst Barker does so $1\frac{1}{4}''$ behind and $1\frac{1}{4}''$ above the meatus. The side of the skull being shaved, and thoroughly cleaned by soap and water, turpentine, and then carbolic lotion, the operation is done with strict antiseptic precautions. The bone is exposed by cutting a semicircular flap with its base downwards, or by a cruciform incision of the soft parts. The periosteum is reflected with the flap, or separately. A small disk of bone is then removed by a trephine (not more than $\frac{3}{4}''$ in diameter necessary). If the abscess is subdural, the dura will bulge into the opening and fluctuation be detected, and on incision the pus will escape: but if the abscess be subcortical, the dura mater is incised, and the lobe explored by either a hollow needle or a small pair of sinus forceps—the latter is preferable, as the needle is liable to be blocked up by brain-tissue. The abscess being found, the opening into it is enlarged by scraping so as to admit a drainage-tube. Barker recommends for this a portion of a silver catheter, so that the split end can be hammered into a flange, to rest on the outside of the scalp. The flap of soft parts is now sewn up, except where the drain-tube comes out, and an antiseptic dressing firmly applied. If antisepsis be carried out, there is very little risk of a hernia cerebri, and the abscess readily heals.

*Cerebellar abscess.*—As already stated, a cerebellar abscess from middle-ear disease is always in the lateral lobe on the same side, generally in its anterior part. The etiology and pathological anatomy are very similar to those of temporo-sphenoidal abscess. Veins pass from the internal ear to the inferior petrosal sinus, and from the mastoid cells to the lateral sinus, whilst to both of these sinuses pass veins from within the lateral cerebellar lobe. So that there is a direct path for inflammatory changes to set up a subcortical abscess. Also, a subdural form of cerebellar abscess may arise as the result of a localised suppurative leptomeningitis over the lateral lobe.

The course of a cerebellar abscess varies: it may slowly enlarge, causing increased general intracranial pressure; later, if not relieved by operation, it will rupture into the meninges, setting up a general septic meningitis. It may rupture early, so that the symptoms hardly at all points to cerebellar abscess; whilst it is very often accompanied from the first by a general meningitis, or sinus-thrombosis, or by both of these, so that its presence is altogether obscured.

Taking, firstly, the case of a slowly-enlarging abscess, this will cause general symptoms precisely similar to those of a slowly-enlarging temporo-sphenoidal abscess; *i.e.*, wasting, slow pulse, normal or subnormal temperature, and, in the latter stages, torpor, deepening into coma. But there will be none of those symptoms peculiar to a temporo-sphenoidal abscess: *i.e.*, aphasia (when abscess is on left side), face and arm paralysis, homonomous hemianopsia, amnesic aphasia (when abscess is on left side), paresis of one or more oculo-motor nerves. And, on the other hand, there will be symptoms indicative of cerebellar lesion, due to the abscess pressing on the middle lobe of the cerebellum. Of these the chief are: reeling gait, with vertigo, and vomiting. Both vertigo and vomiting may occur in a cerebral abscess, but not so markedly; and if either well-marked cerebellar ataxy or severe vomiting be present in a case of otorrhœa, with signs of increasing intracranial pressure and without those peculiar to temporo-sphenoidal abscess, a cerebellar abscess may be diagnosed. The position of headache in cerebellar abscess is either frontal or occipital; its presence is not so characteristic as is tenderness on percussion over the cerebellum. *Optic neuritis* may occur, but rarely as compared with its frequency in cerebral abscess. Two additional symptoms have been insisted on by some observers. McBride says that absence of bone-conduction of sound on the affected side is characteristic of cerebellar as compared with cerebral abscess, and this because the inflammatory tract passes through the internal ear; the path followed however may be by way of the mastoid cells. Also it has been asserted that tenderness over the mastoid, where general symptoms of a brain abscess exist, points to cerebellar abscess. But even though the inflammatory path be through the mastoid cells, yet cario-necrosis of these does not necessarily exist, any more than it does in the tympanic roof in cases of cerebral abscess. An acute cerebellar abscess will so soon rupture into the meninges, setting up an acute septic meningitis, that no sufficiently characteristic cerebellar symptoms will appear to enable a diagnosis of abscess to be made. Again: if cerebellar abscess be complicated, from the first, by the presence of a general septic meningitis or of a sinus-thrombosis, it will not be possible to diagnose its presence.

Thus it would appear that it is possible to diagnose only cases of uncomplicated slowly-enlarging cerebellar abscess.

The procedure to be followed in opening a cerebellar abscess is similar to that in the case of cerebral abscess. It is necessary to avoid the lateral sinus, and to do this Barker advises trephining 1″ below Reid's base-line, and $1\frac{1}{2}$″ behind the auditory meatus.

